# Association of American Medical Editors

**Published:** 1884-06-20

**Authors:** 


					﻿EDITORIALS AND MISCELLANEOUS.
ASS O CIA TION of AMERICAN MEDICALEDITORS.
This Association held its annual meeting at Washington City on the 5th
of may. Dr. Conner delivered an address on the “American Medical Journal
of the Future.” The officers elect for the ensuing year are :
Dr. H O. Marey, of Massachusetts— President.
Dr. J. V. Shoemaker, of Pennsylvania—Vice-President.
Dr. H. O. Walker, of Michigan—Secretary.
				

